# Levamisole Induced Agranulocytosis in a Child with Steroid Dependent Nephrotic Syndrome: A Case Report

**DOI:** 10.31729/jnma.8700

**Published:** 2024-08-31

**Authors:** Mohammad Firoz Anjum, Gaurav Kumar Gupta, Jeetendra Bhandari

**Affiliations:** 1 Department of Paediatrics, Patan Academy of Health Sciences, Lagankhel, Lalitpur, Nepal; 2 Department of General Practice and Emergency Medicine, Patan Academy of Health Sciences, Lagankhel, Lalitpur, Nepal

**Keywords:** *agranulocytosis*, *immunomodulator*, *levamisole*, *steroid-dependent nephrotic syndrome*

## Abstract

Levamisole has been used as an effective immunomodulator. Levamisole has been used for nephrotic syndrome due to its immunostimulant, immunomodulating, and steroid-sparing effects. Common adverse effects of levamisole are gastrointestinal symptoms (nausea, abdominal cramps), and pyrexia. However, agranulocytosis or pancytopenia is also a rare but life-threatening complication of levamisole. This is a case report of a 5-year-old girl who had steroid-dependent nephrotic syndrome for which she was started on levamisole as per her weight, following which she had falling total leukocyte count levels on every visit. Thus, this case report emphasizes identifying this rare side effect and its management.

## INTRODUCTION

Since 1969, levamisole has been used as an anthelmintic and effective immunomodulator.^1,2^ Levamisole has been used in steroid-sensitive nephrotic.^3^ Levamisole has been used for nephrotic syndrome due to its immunostimulant, immunomodulating, and steroid-sparing effects.^4^ Agranulocytosis is a rare but life-threatening complication of levamisole. Agranulocytosis reduces circulating granulocytes with an absolute neutrophil count (ANC) of less than 500/μL.^5^-^6^

We present a case of a 5-year-old girl who is diagnosed with steroid-dependent nephrotic syndrome (SDNS). She was started with levamisole which led to agranulocytosis. This case report provides insight into the rare condition caused by levamisole and its management.

## CASE REPORT

A 5-year-old female child was diagnosed with nephrotic syndrome three months back, for which she was treated with a tablet of prednisolone (2 mg/kg/day daily for six weeks and 1.5 mg/kg/day for the next six weeks) and discharged after attaining remission. She had one episode of relapse one month back while she was on an alternate-day regime of Tab. Prednisolone, for which she was again treated with Tab. Prednisolone daily dosing till remission and discharged on an alternate-day regime of Tab. Prednisolone after 12 days. This time, she presented again to the emergency department (ED) with generalized body swelling. She was on Tab. Prednisolone 1.5mg/kg/day every alternate day before this presentation. Her white blood cell count (WBC) then was 20500 cells per microliter (neutrophil: 80%, lymphocyte: 20%) with an ANC of 16400 cells per microliter. She was started with Tab. Levamisole (2.5 mg/kg) every alternate day and discharged after attaining remission and was advised for follow-up in 2 weeks but was missed to follow-up. After two months, she was again admitted with a complaint of fever for three days with no localizing sign. At the time of her admission, her WBC was 8100 cells per microliter (neutrophil: 2%, lymphocyte: 72%, monocyte: 28%), and her ANC was 162 per microliter. She was started ceftriaxone and levamisole, and prednisolone was continued as per her weight-appropriate previous dose. After three days of this admission, her WBC was 3200 cells per microliter (neutrophil: 35%, lymphocyte: 64%, eosinophil: 1%) with ANC 1120 cells per microliter. At discharge, after six days of admission, her WBC was 4400 cells per microliter (neutrophil: 25%, Lymphocyte: 71%, Monocyte: 4%) with ANC cells 1100 cells per microliter. She was discharged with Tab. Levamisole and Tab. Prednisolone.

After two months, the patient presented again with complaints of fever (unrecorded) for five days, loose stool (7-8 episodes per day) for two days, and abdominal pain (periumbilical) for two days. Her vitals at presentation were within normal values (i.e., temperature: 98.3^o^ F, blood pressure: 100/70 mm of Hg, pulse: 85 per min). She had no signs of dehydration. Abdominal examination and other systemic examinations revealed no abnormality in the patient. During her presentation, she was on Tab. Levamisole (2.5 mg/kg) every alternate day, Tab. Enalapril (0.5 mg/kg/day) twice daily and Tab. Prednisolone (1.5mg/kg) every alternate day. Her blood investigation showed a WBC of 2900 cells per microliter (neutrophil: 8%, lymphocyte: 74%, monocyte: 18%) with ANC 232 cells per microliter, C-reactive protein: 47 mg/L, and normal renal function test. Urine and stool microscopic examination showed no sign of infection. Urine culture showed no growth. Urine albumin was 2+. The stool for hanging drop was negative. In view of levamisole-induced granulocytopenia, her levamisole was stopped, and she started on a stress dose of hydrocortisone (100/m^2^/day). She was admitted to the children's ward for seven days. At the time of discharge, she was prescribed a previous dose of Tab. Prednisolone and Tab. Mycophenolate Mofetil 25 mg/kg/day.

At discharge, her WBC was 3600 cells per microliter (neutrophil: 25%, lymphocyte: 73%, monocyte: 2%) with ANC 900 cells per microliter. Initial follow-up after a week her CBC was 5400 cells per microliter (neutrophil: 28%, lymphocyte: 63%, monocyte: 8%, eosinophil: 1%) with ANC 1512 cells per microliter. After a month during her follow-up, her CBC was 6400 cells per microliter (neutrophil: 58%, lymphocyte: 38%, monocyte: 2%, eosinophils: 2%) with ANC of 3712 cells per microliter.

## DISCUSSION

Nephrotic syndrome is characterized by the appearance of proteinuria (>1 g/m^2^/day), hypoalbuminemia (<25 g/L), generalized edema, and normal glomerular function.^7^ Patients with at least two relapses during treatment with alternate-day steroids or within 14 days after stopping steroid treatment are classified as SDNS.^8^ In a longitudinal study, South Asians had a higher incidence of nephrotic syndrome, which was 15.8/100,000, and an incidence rate ratio of 6.61 compared with Europeans.^9^ A double-blinded randomized control trial in 99 children with SDNS and frequent relapsing nephrotic syndrome (FRNS), where treatment with either levamisole or placebo was given, found there was a significantly longer time to the first relapse in patients treated with levamisole compared to the placebo group.^10^

Common adverse effects of levamisole are gastrointestinal symptoms (nausea, abdominal cramps), and pyrexia.^5^ In a study of 1391 cases of adverse effects of levamisole, 51 cases had leukopenia or neutropenia.^7^ Leukopenia related to the use of levamisole was spontaneously reversible after discontinuation of the treatment.^10^ This was seen in our case too. Her WBC count and neutropenia were also improved in one week, which were further enhanced in the next week. In many cases, agranulocytosis seems to occur within the first two months following levamisole treatment; however, certain cases have demonstrated that agranulocytosis might occur after interruption or long-term treatment.^5,6^ This was similar in our case. She developed neutropenia within two months of starting levamisole. This signifies that a longer duration of treatment with levamisole is required to develop agranulocytosis.

The exact mechanism underlying levamisole-induced neutropenia remains incompletely understood.^6^ Levamisole is believed to create antigen-antibody complexes that deposit on neutrophil surfaces, leading to complement activation and cytolysis. Additionally, it enhances T-cell activation, proliferation, neutrophil mobility, adherence, and chemotaxis.^6^ Furthermore, levamisole acts as a hapten, promoting the production of antibodies against granulocyte antigens, which triggers an immune response resulting in leukocyte destruction.^8,10^ Another hypothesis suggests that levamisole may serve as a substrate for myeloperoxidase, forming reactive metabolites that could stimulate autoimmunity.^7^

Agranulocytosis or pancytopenia is a rare but life-threatening complication of levamisole. So, its awareness and early identification are crucial for a better prognosis.
